# Conceptual Understanding and Conceptual Change Through Human AI Collaboration in Science Education: An Integrated Conceptual Framework from a Systematic Literature Review

**DOI:** 10.12688/f1000research.186991.1

**Published:** 2026-08-08

**Authors:** Riski Muliyani, Andi Suhandi, Eka Cahya Prima, Muslim Muslim, Yudi Kurniawan, Dhita Rismayani Priatna

**Affiliations:** 1 Department of Science Education, Universitas Pendidikan Indonesia, Bandung, West Java, Indonesia; 2 Department of Chemistry Education, Universitas Katolik Parahyangan, Bandung, West Java, Indonesia

**Keywords:** Human AI Collaboration; Conceptual understanding; Conceptual change; Misconception; Systematic reviews.

## Abstract

Human Artificial Intelligence (Human AI) Collaboration in science education is gaining momentum and more research is required on how it can facilitate students’ conceptual understanding and conceptual change. Currently, most studies on AI in science education investigate the use of one particular tool and its effects on learning. However, it is the learning mechanisms that are triggered by these tools that help develop knowledge. This systematic literature review was conducted by performing a systematic search within the Scopus database which was last updated on 15 June 2026. In line with the PRISMA 2020 guidelines, a total of 28 empirical studies research were analyzed. These studies of empirical research were published between 2023 and 2026. Six roles of AI supporting the students’ conceptual understanding as well as five mechanisms supporting the students’ conceptual change were identified. Five interconnected learning mechanisms were derived from the analyzed studies, which are presented in an integrated conceptual framework. The framework is based on the theoretical framework of constructivism, the views on learning of the sociocultural theory, the approach of inquiry based science learning as well as on the metacognitive theory. Within the framework AI functions as a learning partner. The framework illustrates how, within science learning environments that are supported by AI, students continuously construct, evaluate and refine their scientific knowledge in order to achieve deeper understanding.

## 1. Introduction

Advances in Generative Artificial Intelligence (Gen AI) and Large Language Models (LLMs) have recently taken center stage in Intelligent Technologies for Education. While past Intelligent Technologies for Education supported learners to retrieve information, to generate content, or to engage in automated tutoring, today’s sophisticated Artificial Intelligence (AI) systems support learners’ cognition, metacognition, and epistemic processes to construct advanced scientific knowledge ( Kim et al., 2022; Kong et al., 2025). As a result, Human AI Collaboration, or learning in which learners and AI systems engage in a cyclical and continuous process of constructing, evaluating, and improving knowledge, is becoming a new learning paradigm ( Cress & Kimmerle, 2023). That is, AI systems are evolving from being simple instructional aids to being learners’ important epistemic partners who support learners’ reasoning, reflection, explanation, and evidence evaluation at all points in the learning process.

Human AI Collaboration is not merely a new technology to support learning, it transforms our understanding of how scientific knowledge is generated and improved. Rather than just making education more efficient by providing information, giving feedback or supporting learning to solve problems, new technologies support processes of knowledge construction of learners. They generate scientific explanations, visualizations, adaptive scaffolding, dialog for reflection and feedback ( Yan et al., 2024; Zhai et al., 2024). Thus, learning with AI changes our perspective on learning from just transferring scientific knowledge contained in instructional resources to learners, to a collaborative process in which human beings and AI systems jointly and progressively construct knowledge by means of interactions between human thinking and AI supported reasoning ( Tsakeni et al., 2025; Yan et al., 2024).

For the last few decades technology has been developing at a rapid pace and this is also apparent within the area of education. New ways of learning are arising and thus also new ways of teaching within science education. Learning science is more than just learning information; it also requires more than just being able to apply algorithms or to memorize facts. It requires understanding of the world in a scientific way. This process requires learners to construct a coherent conceptual representation of the scientific phenomena that they encounter, to recognize inconsistencies in their prior knowledge, to evaluate competing scientific explanations of natural phenomena, and to reorganize their conceptual structures into scientifically accepted models of natural phenomena ( El Fathi et al., 2025; Pacaci et al., 2024). Hence, the processes of achieving conceptual understanding and that of conceptual change are not two separate ends which learners strive to reach. Rather, these are ongoing processes that learners go through in trying to construct scientific knowledge of the world ( Addido et al., 2022). Research into Human AI Collaboration is an emerging field. Evidence from this emerging field is beginning to reveal the educational value of Human AI Collaboration. It goes beyond increasing learners’ test scores in learning scientific concepts. Rather, it has the potential to transform the ways in which learners can construct, evaluate and transform scientific knowledge in order to make sense of the world. It can help them to identify their own misconceptions, to evaluate evidence, to generate alternative representations and models of scientific phenomena, to engage in reflective dialogue, and to construct mental models of scientific phenomena and then to reconceptualize them through multiple interactions with the AI system ( El Fathi et al., 2025; Zhai et al., 2024).

Although considerable progress has been made in the field of Human AI Collaboration and learning with AI, there is much yet to be understood about learning. Current learning theories of conceptual understanding and conceptual change were developed within the context of human-centered educational settings and thus do not provide enough explanations of learning processes that take place within environments that are mediated by AI. Such environments for Human AI Collaboration are not merely for the transmission of scientific knowledge; they support learners to negotiate meanings, to evaluate AI generated explanations, to compare different representations of identical content, and most importantly, to continuously reorganize their conceptual knowledge by way of their interaction with the AI system. While current learning theories can explain how humans attain conceptual understanding of scientific facts within a human learning environment, they are not sufficient to explain the more complex processes of knowledge construction that take place within Human AI Collaboration environments. A more comprehensive theory of learning is required ( Ding et al., 2024; D. Lee et al., 2021; Pacaci et al., 2024).

A rapidly growing body of research has been conducted on how AI can support education within learning environments by means of various functions ( Almasri, 2024; S. S. Lee & Moore, 2024). Many studies investigate the use of Intelligent Tutoring Systems, conversational agents, adaptive learning, automatically generated learning contents and visualizations as well as formative feedback and diagnostics by means of AI. Most of these technologies are used in isolation so that even though individual technologies have the potential to support learning processes, little is known about the learning processes supported by Human AI Collaboration. Also, it is unclear how different educational mechanisms and technologies interact and how they together can support students in constructing scientific knowledge and in achieving a deeper understanding and conceptual change by means of Educational Mechanisms and Technologies ( Kong et al., 2025; Yan et al., 2024). Thus, currently knowledge on technologies and learning mechanisms is fragmented and mostly lacks a theoretical foundation that explains how single educational mechanisms and technologies interact in order to support students’ conceptual understanding.

Although systematic literature reviews on AI in education have been conducted, they still have limitations. Most studies that focus on different aspects of the use of AI in education, on the instructional value of AI, on the different types of AI technologies that are used in education, on learners’ perspectives on and uses of AI in education, on the ethical issues of the use of AI in education, and so on ( Kamalov et al., 2023; Létourneau et al., 2025), have rarely investigated Human AI Collaboration. In particular, while a number of studies have explained how learning with AI affects learners’ cognitive, metacognitive, and epistemic processes, no systematic literature review has yet developed an integrated theoretical framework that explains how Human AI Collaboration can support learners’ construction of scientific knowledge through the processes of attaining conceptual understanding, achieving conceptual change, and constructing scientific knowledge. Most studies on using AI to support science learning have thus treated the different functions that AI can perform in isolation of each other and thus cannot provide a comprehensive integrated understanding of learning with AI. This review aims at several objectives. These objectives are to be answered by the research questions for this review.

RQ1: How does Human AI Collaboration support students’ conceptual understanding of scientific concepts in science education?

RQ2: How do Human AI Collaborations support students’ conceptual change in science learning?

RQ3: What are the learning mechanisms that connect students’ conceptual understanding and their conceptual change when they learn science by collaborating with AI?

RQ4: What integrated conceptual framework can be developed to describe how Human–AI Collaboration can support students to develop their scientific knowledge through their conceptual understanding and their conceptual change in science education?

## 2. Methods

### 2.1 Study design

The purpose of this study, conducted in the form of a Systematic Literature Review (SLR), was to identify, evaluate and organize empirical studies regarding Human AI Collaboration with the objective of fostering students’ conceptual understanding and supporting their conceptual change in science education. By following a transparent, consistent and replicable procedure, in accordance with the guidelines of the PRISMA model for systematic reviews, the present study aimed at not only providing a mapping of studies, but also at examining the underlying mechanisms that connect conceptual understanding and conceptual change in the context of Human AI Collaboration for science education and to develop a comprehensive conceptual framework ( Page et al., 2021).

### 2.2 Eligibility criteria

To filter the results, criteria for the inclusion and exclusion of studies were defined and applied to the title and abstract as well as the full text screening step based on the PICOS framework (Population, Intervention, Context, Outcomes, and Study Characteristics) ( Brignardello-Petersen et al., 2025). Only studies with students or teachers in a science education setting in which AI was integrated in the learning process were included in the review. The studies had to report on the following learning outcomes: students’ conceptual understanding, students’ conceptual change, students’ misconceptions, and students’ misconceptions remediation. These inclusion and exclusion criteria are listed in detail in Table 1.

**Table 1.** Inclusion and exclusion criteria.

PICOS Criteria	Inclusion	Exclusion
Study Characteristics (S)	• Experimental/quasi experimental research (quantitative, qualitative, mixed methods or design based). • Peer reviewed journal articles and peer reviewed conference papers. • Full text available in English. • Published between 2023 and 2026.	• Literature reviews, systematic reviews, meta analyses, bibliometric studies, editorials, book reviews, conference abstracts, dissertations and theses, and non peer reviewed publications. • Purely theoretical papers without empirical data.
Population (P)	• Any level of learner (primary, secondary, tertiary or informal science education). • Pre service teachers and in service teachers.	• General public samples without educational objectives. • Participants involved in non science learning contexts.
Intervention (I)	• Studies on human AI collaboration in science learning environments where learners interact with AI during their science learning activities. • The AI functions as tutor, coach, mentor, discussion partner, feedback giver, reflection partner, misconception detector etc. as a cognitive scaffold for learning.	• AI system development studies and/or studies that report on AI system’s technical performance. • The use of an AI system solely for administrative tasks (e.g. grading, scheduling, attendance). • AI operating without meaningful learner AI interaction.
Comparator/Context (C)	• Studies on Human AI collaboration are compared to traditional instruction or to non AI learning environments or even without a comparison group within different perspectives on learning processes. • Physics, chemistry, biology, earth science, environmental science, integrated science and/or STEM education.	• Only different AI models, algorithms or system architectures are compared, but not implemented for educational purposes. • Non science educational contexts.
Outcomes (O)	• Evidence of conceptual understanding. • Evidence of conceptual change. • Misconception identification, diagnosis, or reduction. • Knowledge restructuring or conceptual reconstruction. • Degree to which learners develop their understanding of scientific explanations or of scientific reasoning in Human AI collaboration settings.	• Measured outcomes such as users’ satisfaction, technology acceptance, system’s usability, users’ engagement, users’ motivation, and users’ attitudes toward using AI in learning environments without measuring conceptual learning. • The study only presents results regarding the technical accuracy or performance of the AI system developed in the study.

### 2.3 Search strategy

For the literature study a search strategy was developed. The test searches that were conducted with the already known keywords and terms related to the topic of artificial intelligence and conceptual understanding of the search strategy for literature study. A systematic search was then conducted on the database Scopus, a very large and very reliable database of peer reviewed journals, within the scientific area of (experimental and social) education, (natural and social) science and technology. The search string consists of terms that are often used in research on artificial intelligence and the learning outcomes that are relevant to conceptual understanding. The search query that was actually used is: TITLE-ABS-KEY ((“artificial intelligence” OR “generative AI” OR “ChatGPT” OR “large language model” OR “AI chatbot”) AND (“conceptual understanding” OR “concept learning” OR “conceptual change” OR “misconception”)). The literature search was conducted on 15 June 2026. Search results are limited to English language studies published from 2023 onwards in order to identify the most recent relevant evidence for the review. Importantly, studies were selected for this review, in part, because they addressed aspects of Human AI Collaboration, even when that term was not explicitly written. Studies that detailed human AI interactions that supported students’ conceptual understanding and/or addressed issues of students’ conceptual change, and their existing and investigated misconceptions and ways of remediation were selected for this review.

### 2.4 Study selection

**Figure 1.**
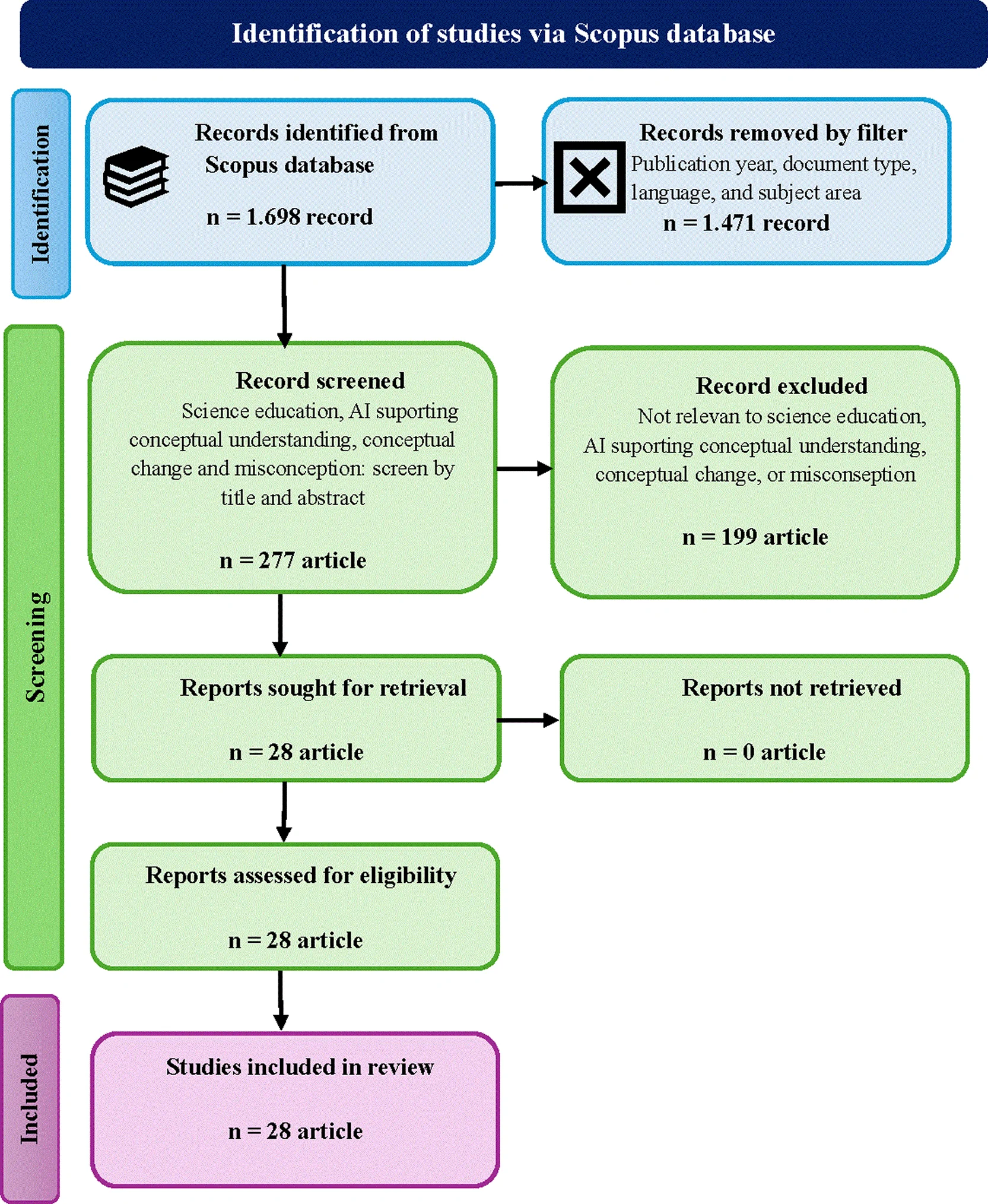
PRISMA flow diagram of the study selection process.

Initially the articles, retrieved through the Scopus search, were downloaded from the Scopus website as a CSV format. Then these articles went through a three step process of screening: title screening, abstract screening and full text review. Articles were excluded on the basis of title screening if they dealt with topics which were clearly not relevant to education and/or did not contain any aspects of AI supported learning. The abstracts of the articles which had not been excluded on the basis of title screening were then screened against the inclusion and exclusion criteria for relevant studies on Human AI Collaboration and their implications for learning and teaching as well as for studies dealing with topics related to conceptual understanding, conceptual change and misconceptions and their remediation. In case of doubt potentially relevant articles were read in their entirety as part of the full text review in order to check whether all inclusion and exclusion criteria were met. Due note was taken of three aspects, namely (1) the type of human AI interaction studied in a particular article, (2) the educational setting in which an AI system was implemented, and (3) the learning outcomes in terms of the learners’ conceptual understanding, their experiences of conceptual change, their identification of and their remediation of misconceptions. The study selection process is presented in the PRISMA flow diagram ( Figure 1).

### 2.5 Data extraction

After selecting the relevant studies, we extracted data from the studies and organized them in an Excel file with four main parts of information. The first part contains information about the studies, i.e., level of participants, scientific domain, type of study and type of AI used in the study. The second part contains information about Human AI Collaboration in the studies, i.e., the role of AI in education, type of human AI interactions and learning activities in which human and AI collaborate to facilitate learning. The third part contains information about the specific conceptual learning outcomes that were measured in the studies, i.e., students’ understanding of specific concepts, level of conceptual change, students’ ability to recognize their own and peers’ misconceptions and students’ ability to remediate these misconceptions. Finally, the fourth part of information that we extracted contains information about the theoretical foundations of the studies and about the learning mechanisms that are used in order to support students’ learning, i.e., feedback, scaffolding, coaching and scripting.

### 2.6 Data analysis and synthesis

This analysis was carried out by means of thematic coding by the author, based on the above stated framework. The selected articles were analyzed with respect to four criteria: (1) form of Human AI Collaboration, (2) learning outcomes in terms of conceptual content, (3) learning mechanisms which promote the development of conceptual content, and (4) theoretical frameworks which are used in order to implement AI in science education. In an iterative process, the findings from the single studies were analyzed using the aforementioned thematic coding. The emerging categories and themes were compared within and across the studies. In addition, the results from the single studies were summarized using descriptive statistics regarding the characteristics of the included studies as well as the applied AI technologies and the forms of Human AI Collaboration. A narrative synthesis of the coding results was conducted to identify recurring patterns, interthematic relationships, and research deficits. The results of the analysis were used to develop a conceptual framework outlining the relations of Human AI Collaboration, learning mechanisms, conceptual understanding, and conceptual change of conceptual understanding in science education.

## 3. Result

### 3.1 Characteristics of the review studies

The present review includes 28 studies and in detail describes the current research on Human AI Collaboration in science education. A synthesis of studies was conducted and a study characteristics table was set up and analyzed with respect to the following criteria: year of publication, students’ educational level, science domain, AI technology used and research design. The trends of Human AI Collaboration in order to support students’ conceptual understanding, conceptual change and to identify and to remediate students’ misconceptions in science education are described in the present review. A summary of the characteristics of the reviewed studies is given in Table 2.

**Table 2.** Characteristics of the included studies.

Characteristics	Categories	n	%
Publication Year	2023	3	10.71
	2024	7	25
	2025	11	39.29
	2026	7	25
Education Level	Primary Education	4	14.29
	Secondary Education	5	17.86
	Higher Education	10	35.71
	Teacher Education	7	25
	Mixed/Professional	2	7.14
Science Domain	Physics	9	32.14
	Chemistry	3	10.72
	Biology	2	7.14
	Environmental and Earth Science	7	25
	Integrated Science/STEM	7	25
AI Technology	LLMs & Generative Chatbots	14	50
	Machine Learning & AIoT	5	17.86
	Intelligent Tutoring Systems & Adaptive AI	4	14.29
	Visualization, Audio & Metaverse	5	17.85
Research Design	Mixed Methods Research	8	28.57
	Design and Development Research	6	21.43
	Experimental Research	5	17.86
	Qualitative Research	4	14.28
	Other Designs	5	17.86

### 3.2. Human AI collaboration for supporting conceptual understanding

We reviewed studies where different AI technologies were used to support Human AI Collaboration and students’ conceptual understanding. Our synthesis showed that Human AI Collaboration supports students’ scientific understanding by constructing, refining, evaluating, and reorganizing scientific knowledge with the help of AI tools that function as a cognitive partner. Human AI Collaboration more than just providing information to students, supports their conceptual understanding by mediating their cognitive and metacognitive processes in developing their conceptual understanding. Our synthesis of the reviewed studies led to the identification of six different but complementary roles of Human AI Collaboration ( Table 3).

**Table 3.** Human AI collaboration for supporting conceptual understanding.

Human AI Role	Function	AI Technologies Used	Conceptual Understanding Outcome	Representative Studies
AI Tutor and Cognitive Scaffolding	Provides adaptive guidance and problem solving support	Rule based chatbot, GPT-4, MyBotBuddy, GPAiM (GenAI agents)	Enhanced conceptual understanding and higher order thinking skills	Khor, E. T., & Chan, L, 2025; Lee, et al., 2023; Lizano-Sánchez et al., 2025; Wu et al., 2026
Inquiry Facilitator and Problem Solving Partner	Exploring data, conducting experiments and modeling of natural phenomena	Teachable Machine, Node RED, XAI (LIME), IoT platforms, Python (Astroquery)	Concept understanding is transferred to computational models in order to deepen practical as well as conceptual understanding	Chookaew et al., 2024; Marín Díaz, 2025; Serik et al., 2025; Zha et al., 2025
Feedback and Assessment Assistant	Provides immediate feedback and formative assessment	ITAS (hybrid scoring system), AI adaptive systems, LLM based evaluation	More accurate identification of students’ conceptual difficulties and improvement in their conceptual performance	Brückner et al., 2026; Kurniawan et al., 2024
Visualization and Explanation Assistant	Presents abstract concepts through multimodal representations	Bing AI, Ideogram, ChatGPT, Claude, TTS tools	Improved comprehension of complex scientific concepts	Clark, 2023; De Souza et al., 2024; Dmitrenko, 2025
Dialogue and Reflection Partner	Facilitates dialogue and metacognitive reflection	ChatGPT, DeepSeek, Teachable Machine (AMAIL framework)	Development of deeper mental models and conceptual reasoning	Kaharu et al., 2026; Relmasira & Donaldson, 2025
Co Creator and Instructional Design Partner	Supports collaborative instructional design and knowledge production	ChatGPT, Gemini, Jupyter AI	Deepened conceptual understanding and improved learning organization	Elshall & Badir, 2025; Gonçalves Costa et al., 2024; Kim, W. J. 2026.

Several functions were assigned to AI in the reviewed studies and as before they are of a pedagogical nature. They are not linked to any single technology and include functions of a tutor, supporting learners in their constructive processes and providing them with adaptive scaffolds. Also discussed were the function of an inquiry facilitator allowing students to deal with scientific issues in a problem solving manner, functions of feedback and assessment as well as of visualization and functions for visualization and explanation of given contents and for enabling dialogue and reflection. Finally there is the function of a co creator: as well as supporting single learners in their knowledge production AI also supports learning in teams. Its use supports the production of knowledge and the design of learning as well as of teaching. The roles for AI presented above thus support the learners’ knowledge construction by backing the various processes instead of single learning tasks.

Most importantly for us, however, the effectiveness of Human AI Collaboration is determined more dependent on the quality of learners’ interactions with AI than on the degree of technical sophistication of the employed technology. While studies have reported generally positive learning outcomes with a variety of forms of AI, learners have been enabled to use intelligent tutoring systems, generative AI, adaptive learning environments as well as a variety of different visualizations to build scientific knowledge and to complete school tasks ( Nguyen et al., 2024; Vaccaro et al., 2024). Thus, various technologies can be used to support essentially the same pedagogical functions, thus functioning primarily as learning partners for Human AI Collaboration. AI should therefore not be confounded with information ( Jose et al., 2025; Kim et al., 2022; Yan et al., 2024).

A key finding from the synthesized material is that the focus of current research on use of AI for science learning has shifted from an assessment of short-term acquisition of science concepts to a more continuous construction and refinement of a learner’s scientific model of the world for science concepts over time ( Hudson et al., 2023; Zhu et al., 2022). Most current research utilizing AI in order to support learning of science concepts involves the use of adaptive tutoring by the AI system. However, a growing body of research is now supporting the use of AI for a number of other functions in support of learning of science concepts. For example: (a) support for scientific inquiry; (b) support for learners’ reflection on learning science; (c) support for learners’ argumentation regarding science concepts; and (d) support for learners’ collaborative meaning making regarding science learning ( Katsenou et al., 2025; Yusuf et al., 2025).

### 3.3. Human AI Collaboration for Supporting Conceptual Change

An analysis of the studies, investigating students’ conceptual change of their understanding when collaborating with AI, revealed five strategies that effectively support students to identify, evaluate and change their own conceptions. These five strategies are presented in Table 4.

**Table 4.** Human AI collaboration for supporting conceptual change.

Human AI mechanism	Function	AI Technologies used	Conceptual change outcome	Representative studies
Misconception Diagnosis and Pattern Detection	Identifies learners’ misconceptions and cognitive structures	Machine Learning Ensemble (Multilayer Perceptron, Support Vector Machine, Random Forest), Natural Language Processing, GPT-4, GPT-5	More accurate diagnosis of misconceptions	Braune et al., 2026; Conradty & Bogner, 2026; Kökver et al., 2025
Adaptive Scaffolding and Feedback Driven Revision	Provides adaptive feedback and corrective explanations for erroneous concepts	Rule-based Intelligent Tutoring System (Aristarchus), Learning Analytics Dashboard	Knowledge restructuring and enhanced scores in student understanding	Georgiou et al., 2026; Pektaş et al., 2026
Reflective Dialogue and Mental Model Externalization	Helps students explore and externalize their mental models	Bing AI/DALL.E, ChatGPT	Better scientific conceptual coherence and deeper reflection	De Souza et al., 2024; Ding, et all., 2023; Nendauni, 2025
Cognitive Conflict and Affective Monitoring	Leverages cognitive conflicts experienced by students to trigger conceptual change	Affectiva Facial Emotion Recognition, iMotions	Conceptual shift from misconceptions to scientific concepts, accompanied by students’ “aha” or “oh” moments	Ezquerra et al., 2024
Fact Checking and Information Validation	Encourages critical evaluation and analysis of AI generated responses	ChatGPT	Improved scientific literacy and information validation skills	Mennella & Quadros-Mennella, 2024

The reviewed studies examined in what ways Human AI Collaboration can support students’ conceptual change. On the basis of five different types of AI technologies five different mechanisms for fostering the five mentioned steps for giving given conceptions a more accurate form are identified and described in Table 4. These five mechanisms supporting students in identifying, evaluating or even changing their given conceptions step by step for developing more accurate scientific explanations for a given problem of science learning in general support a process of knowledge restructuring. Thus, in addition to identifying their given misconceptions the five different types of AI tools support learners in developing more accurate scientific explanations for a given problem of science learning instead of being used in isolation as a variety of separated educational tools.

In addition to identifying various forms of technology used to support various functions in order to bring about conceptual change through learners’ active processing of learning processes (e.g. diagnose misconceptions and support feedback, adaptive feedback and reflection, as well as information evaluation), the results reveal that the greatest value of AI in the context of education pertains to its pedagogical potential. The current state of research in Human AI Collaboration can be synopsized by the so-called corrective perspective. First, the AI system recognizes mistakes by learners and then the AI system supports learners in their learning process by means of adaptive feedback that replaces learners’ misconceptions with corresponding scientific conceptions ( Ding et al., 2024; El Fathi et al., 2025; Pacaci et al., 2024). Such an AI system supports learners to attain higher levels of conceptual understanding. However, from a theoretical point of view, current approaches to Human AI Collaboration only explain parts of processes of conceptual change because they do not sufficiently account for learners’ active construction of scientific knowledge while they are engaged in learning with an AI system, whereas error correction by the AI system is predominantly in the focus of current approaches to Human AI Collaboration.

New research on AI is increasingly starting to consider it from a broader knowledge construction perspective. Thus, as opposed to focusing on its use for diagnosis or for correction, AI is increasingly being viewed as a learning partner with whom students can engage in reflection, dialogue, argument, meaning making and knowledge construction ( Sánchez Muñoz et al., 2025). Conceptual change as a result of such AI mediated learning is a gradual process of examination, of justification, of refinement and of restructuring of scientific knowledge, a process that takes place while students are interacting with the technology that supports their learning.

While there are an increasing number of studies that investigate how humans and AIs can support each other in learning, an in depth analysis of such collaboration is still rare in the literature. While misconception diagnosis and feedback are still the focus of most studies, the potential of AIs to support students in their reflection, in their collaborative reasoning and in their knowledge construction based on evidence, is not yet explored enough. Thus, in the future, research on Human AI Collaboration in learning has to move from technology based interventions to design of environments that support conceptual change as a process of scientific knowledge construction over time.

### 3.4. Mechanisms linking conceptual understanding and conceptual change

In contrast to the two distinct learning objectives of conceptual understanding and conceptual change within Human AI Collaboration, these two objectives are part of a continuum of learners’ processes of developing their scientific understanding when collaborating with AI. Combining the results from Tables 3 and 4, five interconnected learning mechanisms of learners’ processes of developing, examining and restructuring their scientific concepts were identified (see Table 5). The processes of developing, examining and restructuring of learners’ scientific concepts form a continuum leading from their first conceptual understanding to more refined or even completely replaced concepts by more adequate scientific explanations during Human AI Collaboration over time.

**Table 5.** Learning mechanisms of human AI collaboration supporting conceptual understanding and conceptual change.

Learning Mechanism	Contribution to Conceptual Understanding and Conceptual Change	Limitations in the Literature
Adaptive Support (Scaffolding and Feedback)	Helps learners to first come up with ideas and to correct mistakes by means of learning support which is given in the form of hints and feedback.	While the majority of studies on formative feedback on test results concentrate on error correction and in the long run on test scores, only a few studies investigate the matter of long term conceptual change.
Knowledge Exploration and Construction	Supports learners to build up mental models of their knowledge and to understand concepts by problem solving, visualization or different representations of the knowledge to be learned by the learner.	Most of the studies that have been mentioned above above follow learners in order to investigate the final results of their understanding of scientific concepts, whereas an in depth study of the developmental processes of their understanding is rarely carried out.
Reflection and Dialogue	Students can also explain, evaluate or even change their own ideas or understanding by having a dialogue with the AI or by reflecting on its output.	Even though nowadays so called reflective dialogues are more and more often used in educational processes, they are mostly treated as additional support in AI based instructional design processes.
Cognitive Conflict	Cognitive conflict and its role in bringing about learners’ knowledge restructuring by recognizing dissonance between their initial understanding and scientific explanations.	Cognitive conflict is mostly used in isolation from other strategies such as reflection and argumentation in educational design.
Information Evaluation and Validation	Aids students to verify facts and answers generated by the AI system and more importantly teaches them to evaluate information from any source.	Most studies on using AI in science education treat AI as mainly giving answers to questions posed by the student and only secondarily as learning partner which the student can use to verify information and together with the AI to co construct scientific meaning and evaluate sources of information for accuracy.

These five functions are part of a learning process. Firstly, learners get to know new things with the help of adaptive support and feedback. Then they work more on the knowledge they already have acquired with the help of knowledge exploration, visualization, problem solving and various ways of presenting scientific content. Later on in the learning process, learners can reflect on their own learning process by means of discussion and the explanation of their own ideas. They question their own ideas and improve them by discussing with others. In order to make learners aware of differences between their existing conceptions and the scientific explanations, the cognitive conflict that occurs is a crucial function. The functions of information evaluation and validation are the last functions in the learning process. In these functions learners check the AI generated responses and other sources of evidence that they have found by themselves. After that they integrate the new scientific knowledge that they have acquired into their existing understanding of the corresponding scientific content. In this way, conceptual change is a continuous process of learning that takes place step by step by means of various learning activities. Several forms of technology support the same processes of learning. Thus, Intelligent Tutoring Systems, generativeAI, machine learning, learning analytics, data visualization, and conversational AI Systems all support learning processes that remain consistent across technologies. So, supporting Human AI Collaboration efficiently for learning depends more on how these several forms of technology can support learning than on the technology itself.

The current research is one sided, as the majority of studies and research on adaptive technologies for learning, on misconception diagnosis, and on feedback, are rooted in the traditional corrective view of learning for conceptual change ( Katsenou et al., 2025; Kim et al., 2022). These studies and other similar studies offer insights on how students can be supported in order to achieve higher conceptual accuracy in learning scientific concepts, however, they only outline a part of the learning process for achieving such concepts, as students are not supported in constructing their own scientific knowledge by means of inquiry, reflection, dialogue and collaborative reasoning.

A further finding from this review is the contrast between the current research on conceptual change on the one hand, and recent Human AI Collaboration studies on the other hand. Theories on conceptual change describe a slow process in which learners and their learning environments incrementally add to the learners’ existing knowledge by means of reflection, discussion, argumentation, etc. Instead of this, recent studies on Human AI Collaboration still view the AI system mainly as a diagnostic or corrective tool that supports the learners in case they do not arrive at a correct solution. In the findings from this review, however, the AI system is viewed as a learning partner that supports the learners in their processes of exploring ideas, in comparing their own explanations of phenomena, in evaluating evidence for claims, and in developing a scientific understanding of phenomena in a step by step manner. Future research on Human AI Collaboration therefore should not be restricted to the analysis of single mechanisms that are used by AI systems to support human learning in science classrooms and to study their effects on human learning in given contexts. Rather, future research should also be aimed at analyzing how several of these mechanisms can work together in order to support learners in a long term process of knowledge construction and of conceptual change.

### 3.5. Integrated framework of human AI collaboration for conceptual understanding and conceptual change

**Figure 2.**
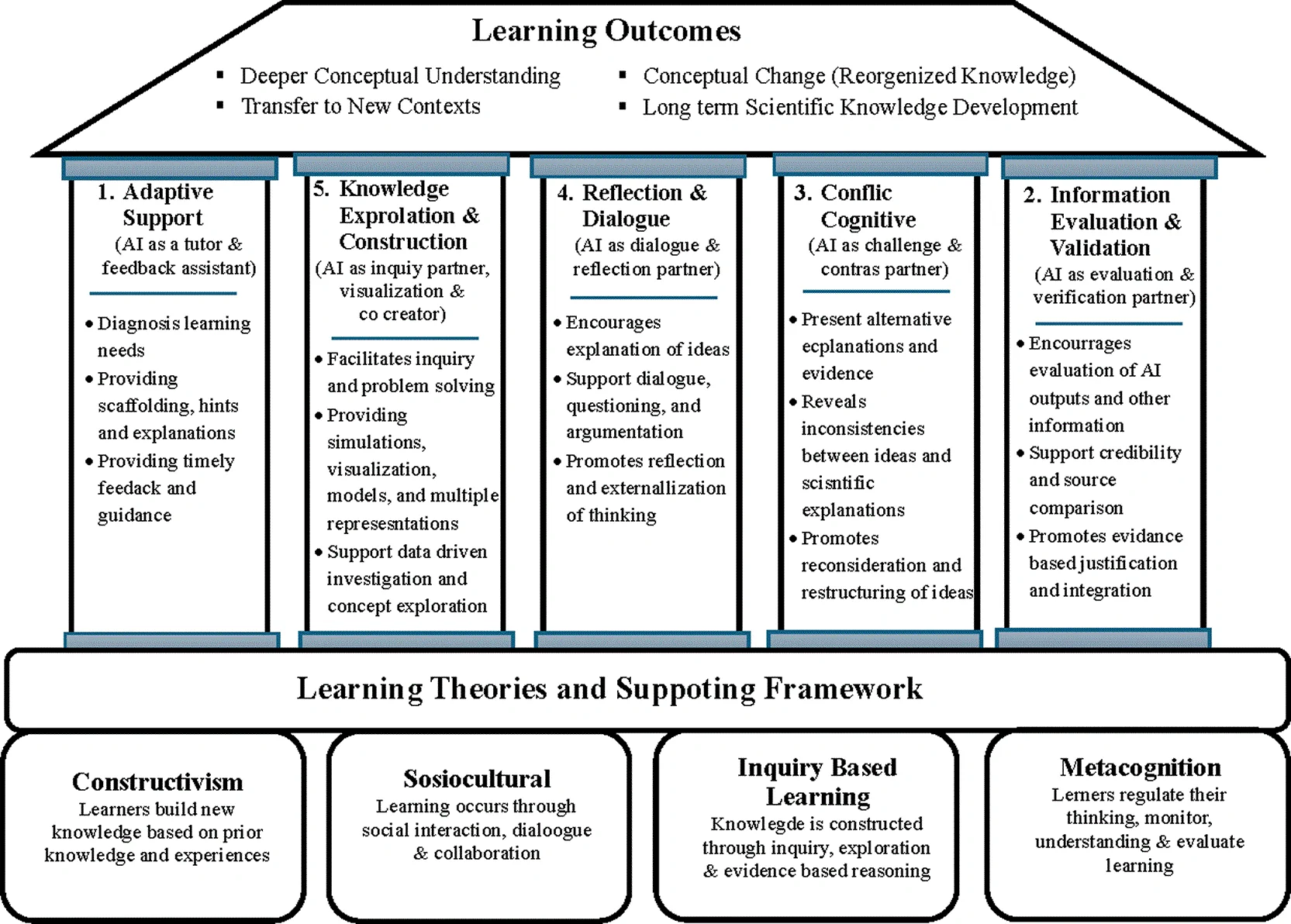
Framework of Human AI Collaboration.

The functions for Human AI Collaboration, which are supported by a variety of AI functions, support both the conceptual understanding and the conceptual change of students. For the support of Human AI Collaboration for joint knowledge construction, a large number of studies on tutoring systems, adaptive feedback systems, dialogue systems, AI based visualization systems, and so on, were reviewed. The effects of the functions for Human AI Collaboration were studied in a number of cases, but in each case, the functions were studied in isolation. Therefore, knowledge about the effects of the functions for Human AI Collaboration is currently fragmented and insufficient to explain the interaction of the different learning processes that support the long term development of students’ scientific knowledge in detail. An integrated conceptual model was developed on the basis of Tables 3-5 to explain how the different AI supported learning mechanisms support the transition of students from their conceptual understanding to their conceptual change ( Figure 2).

Our framework is based on four main learning theories. The first of these is Constructivism. In AI supported science learning children can construct a scientific understanding of the world by using their prior knowledge and learning new information ( Nguyen et al., 2024). In doing so they go through different phases of cognitive conflict as their current conceptions are challenged by real world data. The second learning theory is Sociocultural Theory. This theory explains how children learn by interacting with their environment, through dialogue and the shared meaning making of learners, teachers, peers and now technology ( Wheaton et al., 2024). The third learning theory that we used is Inquiry Based Learning. Children develop a scientific understanding of the world by carrying out an investigation into a question or problem. In doing so they use evidence, modeling and solve problems in order to arrive at a conclusion ( Morris, 2025). The last learning theory that we used is Metacognitive Theory. This theory explains how children can monitor, evaluate and control their own learning while using AI generated explanations ( Tsakeni et al., 2025). Together these four learning theories provide a coherent explanation of the different processes that take place during AI supported science learning. Children’s conceptual understanding develops progressively over time through a number of different processes instead of just one single event.

In order to support learning in a sustainable way, five mechanisms for learning interact in the process of knowledge construction. At first adaptive support for learning is generated in order to establish a first understanding of the subject matter under study. This basic understanding is then in turn consolidated during the learning process by means of knowledge exploration and knowledge construction by means of inquiry, visualization, modeling and the use of multiple representations for one and the same content. In the process of learning about his or her own learning processes by means of reflection and dialogue, the learner can take a closer look at his or her own thinking. Therefore, he or she is more likely to experience a cognitive conflict when scientific evidence confronts his or her existing conceptions ( Pacaci et al., 2024; Pektaş et al., 2026). However, instead of merely changing single misconceptions, the learner is able to reorganize his or her entire existing knowledge structure as a result of this conflict ( Shih et al., 2026). In the process of learning, information evaluation and validation are crucial. Learners check their AI generated responses as well as other information in a critical manner ( Walter, 2024). They integrate the resulting scientific explanations that have been generated in this process step by step into their already existing knowledge structures. This stepwise development of the conceptual understanding of a learner leads to so called conceptual change after several cycles of the mentioned mechanisms for learning.

The proposed framework for Human AI Collaboration builds upon current research by identifying a number of as yet underdeveloped aspects. In contrast to previous studies on Human AI Collaboration, which have investigated adaptive tutoring, feedback, visualization, dialogue, or even misconception diagnosis as separate instructional functions and their short and long term effects on learning outcomes, our framework describes how these functions can function as a number of interrelated learning mechanisms that support the learners’ ongoing construction of scientific knowledge ( Ji et al., 2023; Thomas et al., 2024). While previous studies treat AI primarily as a teaching aid for correcting students’ misconceptions, our framework views the AI system as a learning partner. In contrast to the traditional view of conceptual change as a process of replacing students’ incorrect conceptions with correct scientific conceptions, which is refined and restructured in detail through their prolonged interactions in AI supported learning environments, the learners’ scientific understanding is in the long run refined and restructured in detail through their prolonged interactions in AI supported learning environments.

This framework also points out several avenues for future research. As already is the case with adaptive feedback and with correcting misconceptions, research on reflection, reflection, dialog, and cognitive conflict as processes of learning that are interwoven and as yet too not fully researched when using AI for learning, will be very important. Most studies on the use of AI in learning so far investigate one function of the AI system in isolation and then follow its use in one learning session or in several learning sessions ( Bauer et al., 2025). Instead, it is more important to investigate how in the long run several functions support learning when used in combination. The integrated framework that we presented here can be used as a starting point for the design of learning environments in which AI supports learning of science and in which learners construct, evaluate, and change scientific explanations for real world phenomena while acquiring knowledge of science.

## 4. Conclusion

Human AI Collaborations in science education is a field of research that is rapidly evolving and not yet fully understood. While there are many excellent studies that focus on how AI can support students in achieving their learning goals for scientific learning, there is a severe lack of in depth studies on how Human AI Collaborations can support students’ conceptual understanding and their process of conceptual change in science. Our review aimed to answer the question of how the AI system functions as a collaborative learning partner in order to support the continuous construction, evaluation, and refinement of students’ scientific knowledge about science. As a result of our review, it seems to be more appropriate to treat the processes of students’ conceptual understanding and their conceptual change as a matter of fact. Within the given framework of the learning environment, these processes are brought about by a variety of learning mechanisms rather than by a number of separate teaching methods that are intended to achieve a number of specific goals.

An integrated conceptual framework for studying Human AI Collaborative Learning in Science Education is proposed here. The framework outlines relationships between several learning theories, a set of mechanisms that can be supported by AI in order to foster Human AI Collaborative Learning, and ways by which knowledge is constructed during Science learning. This framework can serve as a groundwork for future research on Human AI Collaborative Learning in Science, for the design and implementation of environments where Humans and Artificially Intelligent agents collaborate in learning, and for the study and design of pedagogical approaches where AI functions as a learning partner for the processes of inquiry, reflection, critical evaluation and the continued construction and development of scientific knowledge during the scientific learning process.

## Use of AI assisted technology

The authors of this work used Gemini during the preparation of this work for the translation and improvement of the academic language from Indonesian to English.

## Data Availability

The completed PRISMA 2020 Checklist and Flow Diagram for Human AI Collaboration for Supporting Conceptual Understanding and Conceptual Change in Science Education: A Systematic Literature Review has been uploaded to Zenodo and published under Creative Commons Zero v1.0 Universal (CC0) license. Checklist and Flow Diagram can be downloaded online at https://doi.org/10.5281/zenodo.21430817 ( Muliyani et al., 2026).
