# Supplementary material for: Conceptual Understanding and Conceptual Change Through Human AI Collaboration in Science Education: An Integrated Conceptual Framework from a Systematic Literature Review
Source: F1000Res. 2026 Aug 8;15:1339. [Version 1] doi: 10.12688/f1000research.186991.1 (PMC13612998; doi:10.12688/f1000research.186991.1)
Supplement: Peer review history

# Reviewer report: Reviewer response for version 1

**DOI:** 10.5256/f1000research.206380.r520683

**Published:** 2026-09-24

**Authors:** Prima Aswirna

**Affiliations:** 1 Universitas Islam Negeri Imam Bonjol Padang, Padang, Indonesia

1. Absence of an Operational Definition for "Human-AI Collaboration"

 The manuscript positions "Human-AI Collaboration" as its central paradigm but fails to explicitly define what constitutes genuine collaboration versus routine AI usage (e.g., intelligent tutoring, automated feedback, or simple prompt-response interactions). Without distinct inclusion criteria for collaborative dynamics, standard AI-assisted learning tools are conflated with collaborative partnerships.

 2. Blurred Distinction Between Conceptual Understanding and Conceptual Change

 The text treats conceptual understanding and conceptual change almost interchangeably at times, despite their fundamental theoretical differences. Conceptual understanding involves constructing and enriching accurate scientific representations, whereas conceptual change entails restructuring, revising, or replacing entrenched misconceptions. The review does not clearly state whether included studies explicitly measured conceptual change or if change was merely inferred from higher understanding scores.

 3. Single-Database Search Bias (Scopus Only)

 Limiting the systematic literature search exclusively to Scopus introduces significant selection bias. Given the interdisciplinary nature of AI in science education—spanning computer science, educational technology, and cognitive science—key databases such as Web of Science, ERIC, IEEE Xplore, Education Research Complete, and PsycINFO should have been included.

 4. Unjustified Temporal Cutoff (2023–2026)

 Restricting the review to studies published from 2023 onwards excludes foundational pre-2023 empirical research on intelligent tutoring systems, automated scaffolding, and conversational agents in science education. The authors provide no methodological rationale explaining why earlier empirical work on Human-AI collaboration was omitted.

 5. Lack of Protocol Preregistration

 The review protocol was not preregistered in public registries such as PROSPERO or Open Science Framework (OSF). Preregistration is a core standard of PRISMA guidelines to prevent post-hoc changes to inclusion criteria and selective outcome reporting.

 6. Absence of Quality Appraisal / Risk of Bias Assessment

 The review does not conduct a formal critical appraisal or risk of bias evaluation of the 28 included empirical studies. Without evaluating study quality (e.g., sample size validity, control group rigor, measurement instrument reliability), the strength of the synthesized evidence remains unverified.

 7. Omission of Inter-Rater Reliability

 The authors state that screening and selection were conducted, but fail to report whether independent dual screening occurred or what inter-rater agreement metrics (e.g., Cohen’s $\kappa$ or percentage agreement) were achieved. This leaves the screening process vulnerable to individual selection bias.

 8. Single-Coder Bias in Thematic Analysis

 Section 2.6 indicates that thematic coding was performed "by the author" (singular). Conducting qualitative coding without a second independent coder or inter-coder reliability checks undermines the trustworthiness and objectivity of the derived themes and mechanisms.

 9. Overly Causal Claims Based on Non-Causal Evidence

 The review claims that "these processes are brought about by a variety of learning mechanisms" (p. 13). Asserting direct causality is methodologically inappropriate because the included studies consist of heterogeneous qualitative, design-based, and quasi-experimental research rather than pooled randomized controlled trials or meta-analyses.

 10. Ambiguous and Unsubstantiated Assertions ("As a Matter of Fact")

 In Section 4, the authors state that "it seems to be more appropriate to treat the processes of students' conceptual understanding and their conceptual change as a matter of fact" (p. 13). This phrasing is scientifically ambiguous and overstates what can be inferred from a synthesis of 28 studies.

 11. Unsubstantiated Introductory Claims

 The opening statement that Human-AI Collaboration "is gaining momentum" is presented as a premise without supporting bibliometric data, publication growth rates, or citation trend analyses.

 12. Theoretical Eclecticism Without Integration Rationale

 The proposed framework combines four major learning theories: Constructivism, Sociocultural Theory, Inquiry-Based Learning, and Metacognitive Theory. However, the paper does not adequately explain how these distinct traditions—which hold differing assumptions about individual cognition versus social interaction—conceptually align or why alternative learning models were excluded.

 13. Undefined Interrelationships Among Framework Components

 Figure 2 presents five learning mechanisms as separate pillars beneath learning outcomes. The manuscript fails to specify whether these mechanisms operate sequentially, iteratively, conditionally, or hierarchically during student-AI interactions.

 14. Undifferentiated Aggregation of AI Technologies

 The synthesis groups widely divergent AI technologies—ranging from rule-based chatbots and intelligent tutoring systems to Large Language Models (LLMs), machine learning models, and IoT platforms—under the single banner of "AI". Because LLMs possess vastly different affordances and hallucination risks compared to rule-based tutors, treating them as functionally equivalent obscures tool-specific impacts.

 15. Anthropomorphic Framing of AI as a "Learning Partner"

 The manuscript repeatedly characterizes AI as a "learning partner" possessing epistemic authority. Framing non-sentient software as an active agent risks misrepresenting computational prompt-response loops as genuine social/collaborative reciprocity.

 16. Numerical and Structural Inconsistencies

 The paper asserts that it identified "six roles of AI" in supporting conceptual understanding and "five mechanisms" supporting conceptual change, but elsewhere presents five learning mechanisms connecting the two. The structural mapping between the 6 roles (Table 3) and the 5 mechanisms (Tables 4 & 5) is left visually and conceptually disconnected.

 17. PRISMA Reporting Discrepancies

 Although the text claims alignment with PRISMA 2020 guidelines, key details (such as detailed exclusion breakdown numbers per screening stage) are omitted from the main text and relegated to an external Zenodo link rather than being fully articulated within the body of the article.

 18. Narrow and Vulnerable Search Query Strategy

 The search string TITLE-ABS-KEY (("artificial intelligence" OR "generative AI" OR "ChatGPT" OR "large language model" OR "AI chatbot") AND ("conceptual understanding" OR "concept learning" OR "conceptual change" OR "misconception")) omits broader terms like "cognitive conflict," "schema restructuring," "adaptive scaffolding," or specific domain terms. Furthermore, searching without domain-specific keywords forced reliance on post-hoc manual filtering to isolate science education studies.

 19. Search Date Ambiguity

 The manuscript states that the Scopus search was "last updated on 15 June 2026". It remains unclear whether this date reflects the initial search or a re-run, leaving the exact timeline and reproducibility of record retrieval ambiguous.

 20. Overrepresentation and Domain Bias Toward Physics

 According to Table 2, Physics accounts for 32.14% of the studies, while Chemistry (10.72%) and Biology (7.14%) are severely underrepresented. The authors do not address how this domain skew impacts the generalizability of their framework across other scientific disciplines with differing conceptual structures.

 21. Methodological Vagueness in Narrative Synthesis

 Section 2.6 mentions that a "narrative synthesis of the coding results was conducted", but fails to define the specific synthesis protocol used (e.g., Popay et al.'s framework for narrative synthesis). This leaves the step-by-step translation from empirical findings to framework components unverified.

 22. Incomplete Categorization in Descriptive Statistics

 In Table 2, under "Research Design," categories such as "Other Designs" (17.86%) are left undefined. Furthermore, criteria for distinguishing overlapping categories (e.g., "Mixed Methods Research" vs. "Experimental Research") are omitted.

 23. Failure to Assess Publication Bias

 The review does not discuss or account for publication bias. Given that studies publishing positive learning gains from novel AI implementations are more likely to be accepted than null results, the review likely overestimates the efficacy of AI in fostering conceptual change.

 24. Lack of Practical and Instructional Translation

 While the article presents a high-level conceptual framework (Figure 2), it provides minimal guidance on how educators or instructional designers can operationalize these mechanisms in actual classroom settings. It lacks practical prompt structures, activity designs, or curriculum integration protocols.

 25. Grammatical Errors and Structural Redundancies

 The text contains several grammatical and stylistic flaws that detract from its academic precision, such as "a total of 28 empirical studies research were analyzed" (p. 1) and repetitive phrasing like "These studies of empirical research." Minor typographical errors in headings (e.g., "Knowledge Exprolation & Construction" in Figure 2) also remain uncorrected.

Are the rationale for, and objectives of, the Systematic Review clearly stated?

Partly

Is the statistical analysis and its interpretation appropriate?

Partly

If this is a Living Systematic Review, is the ‘living’ method appropriate and is the search schedule clearly defined and justified? (‘Living Systematic Review’ or a variation of this term should be included in the title.)

Partly

Are sufficient details of the methods and analysis provided to allow replication by others?

Partly

Are the conclusions drawn adequately supported by the results presented in the review?

Yes

Reviewer Expertise:

Educational Science and AI in Education Science.

I confirm that I have read this submission and believe that I have an appropriate level of expertise to confirm that it is of an acceptable scientific standard, however I have significant reservations, as outlined above.

# Reviewer report: Reviewer response for version 1

**DOI:** 10.5256/f1000research.206380.r520675

**Published:** 2026-09-15

**Authors:** Torang Siregar

**Affiliations:** 1 Education Sciences, Mathematics Education, State University of Padang, Padang, West Sumatra, Indonesia

**Peer Review Report**

 The manuscript presents a systematic literature review (SLR) investigating how Human-AI Collaboration supports students' conceptual understanding and conceptual change in science education. The authors analyzed 28 empirical studies published between 2023 and 2026, identified through a Scopus database search. The review identifies six roles of AI supporting conceptual understanding (AI tutor, inquiry facilitator, feedback assistant, visualization assistant, dialogue partner, and co-creator) and five mechanisms supporting conceptual change (misconception diagnosis, adaptive scaffolding, reflective dialogue, cognitive conflict, and fact-checking). The authors propose an integrated conceptual framework grounded in constructivism, sociocultural theory, inquiry-based learning, and metacognitive theory, positioning AI as a "learning partner" rather than merely an instructional tool. The framework illustrates how students continuously construct, evaluate, and refine scientific knowledge through interconnected learning mechanisms.

**Answers to Review Questions**

**1. Are the rationale for, and objectives of, the Systematic Review clearly stated?**

**Strengths:**

1. The introduction provides a reasonable overview of the growing role of AI in education and the shift from AI as a tool to AI as an epistemic partner.
2. The four research questions (RQ1–RQ4) are explicitly stated and map onto the review's objectives.
3. The authors identify a genuine gap: most existing reviews focus on AI in education broadly, but few examine Human-AI Collaboration specifically in relation to conceptual understanding and conceptual change.

**Weaknesses and Required Revisions:**

1. **The rationale for focusing exclusively on "Human-AI Collaboration" is underdeveloped.** The introduction does not adequately distinguish Human-AI Collaboration from related constructs such as AI-assisted learning, AI-supported learning, or intelligent tutoring. The authors assert that Human-AI Collaboration represents "a new learning paradigm" (p. 3) but do not provide a clear operational definition or criteria for what constitutes "collaboration" versus mere "support" or "assistance." This is a critical conceptual gap that undermines the review's coherence.
2. **The claim that Human-AI Collaboration "is gaining momentum" lacks empirical support.** This is a plausible assertion, but it is presented without citation to bibliometric data, trend analyses, or prior reviews that would substantiate it. The authors should provide evidence (e.g., publication trends, citation patterns) to support this claim.
3. **The gap statement is somewhat overstated.** The authors claim that "no systematic literature review has yet developed an integrated theoretical framework that explains how Human AI Collaboration can support learners' construction of scientific knowledge" (p. 4). While this may be true for the specific intersection of Human-AI Collaboration, conceptual understanding, and conceptual change in science education, the authors should acknowledge related frameworks in the learning sciences and AI in education literature (e.g., knowledge building, computer-supported collaborative learning) that may offer partial integration.
4. **The rationale for excluding studies before 2023 is not justified.** Given the long history of intelligent tutoring systems, adaptive learning, and conversational agents in science education, the decision to restrict the review to 2023–2026 requires explicit justification. The authors should explain whether earlier research was excluded because it did not meet the Human-AI Collaboration criteria or because of a deliberate decision to focus on recent developments.
5. **The objectives of the review could be more precisely articulated.** While the research questions are clear, the introduction does not explicitly state what the review aims to contribute beyond the framework itself—for example, whether it aims to inform instructional design, guide future research, or provide a theoretical synthesis.

**Recommendations:**

1. Provide an explicit operational definition of Human-AI Collaboration and distinguish it from related constructs.
2. Support the "gaining momentum" claim with bibliometric or trend evidence.
3. Justify the 2023–2026 publication window.
4. Clarify the review's intended contribution and audience.
5. Acknowledge related frameworks and explain how the proposed framework extends or differs from them.

**2. Are sufficient details of the methods and analysis provided to allow replication by others?**

**Strengths:**

1. The authors report following PRISMA 2020 guidelines and provide inclusion/exclusion criteria based on the PICOS framework.
2. The search string is explicitly stated.
3. The study selection process is described in three stages (title, abstract, full-text screening).
4. A PRISMA flow diagram is referenced (Figure 1).

**Weaknesses and Required Revisions:**

1. **The review protocol was not preregistered.** The authors do not report whether the review protocol was registered (e.g., PROSPERO, OSF, or another registry). Preregistration is a hallmark of rigorous systematic reviews and should be reported. If the review was not preregistered, this limitation should be acknowledged.
2. **Database selection is unjustifiably narrow.** The review relies exclusively on Scopus. While Scopus is a large and reputable database, a single-database search introduces database-selection bias, particularly in an interdisciplinary area spanning education, learning sciences, computer science, and AI. The authors should either (a) expand the search to include Web of Science, ERIC, Education Research Complete, PsycINFO, IEEE Xplore, or other relevant databases, or (b) provide a strong justification for using Scopus alone and acknowledge this as a limitation.
3. **Inter-rater reliability is not reported.** The authors state that screening was conducted but do not report whether multiple reviewers independently screened studies, how disagreements were resolved, or what the inter-rater reliability was. This is a significant methodological omission. At minimum, the authors should report Cohen's kappa or percentage agreement for screening decisions.
4. **The PRISMA flow diagram is referenced but not provided in the manuscript text.** The authors state that the PRISMA checklist and flow diagram are available on Zenodo, but the flow diagram should be included in the manuscript (or at least summarized in text) to show the number of records identified, screened, excluded, and included, along with reasons for exclusion. The current manuscript does not report these numbers.
5. **Search date ambiguity.** The authors state the search was "last updated on 15 June 2026" (p. 4). It is unclear whether this was the original search date or an update. If it was an update, the original search date should be reported. This distinction is important for reproducibility and for understanding the currency of the evidence base.
6. **Data extraction process lacks detail.** The authors state that data were extracted into an Excel file with four main parts, but do not report:
  1. Who performed the extraction (one author or multiple)?
  2. Whether extraction was piloted or calibrated?
  3. How discrepancies were resolved?
  4. Whether authors of primary studies were contacted for missing data?
7. **Thematic coding process is underdescribed.** The authors state that thematic coding was conducted "by the author" (singular) (p. 7). This raises concerns about bias and reliability. The authors should report:
  1. Whether coding was inductive, deductive, or abductive.
  2. How the coding framework was developed and refined.
  3. Whether a second coder was involved and, if so, how reliability was established.
  4. How themes were derived and validated.
8. **Descriptive statistics are reported but not fully explained.** Table 2 presents frequencies and percentages for publication year, education level, science domain, AI technology, and research design. However, the authors do not explain how studies were categorized (e.g., how "Mixed Methods Research" was distinguished from "Experimental Research") or how many studies fell into each category for certain variables (e.g., the table shows percentages but not always clear n values).
9. **Quality appraisal of included studies is absent.** The authors do not report conducting a risk of bias or quality appraisal of the included studies. While quality appraisal is not always required in systematic reviews of this type, it is strongly recommended and would strengthen the review's credibility. If quality appraisal was not conducted, this limitation should be acknowledged.
10. **Synthesis methods are insufficiently described.** The authors state that "a narrative synthesis of the coding results was conducted" (p. 7), but do not describe how the synthesis was performed, how themes were compared across studies, or how the integrated framework was derived from the synthesis. A more detailed account of the analytical process is needed.

**Recommendations:**

1. Report whether the review protocol was preregistered and provide a registration number or URL if available.
2. Expand the database search or justify the use of Scopus alone and acknowledge the limitation.
3. Report inter-rater reliability for screening and coding.
4. Include the PRISMA flow diagram in the manuscript and report the number of records at each stage, with reasons for exclusion.
5. Clarify the search date(s) and whether the search was updated.
6. Provide more detail on data extraction and thematic coding, including who performed these tasks and how reliability was ensured.
7. Report quality appraisal of included studies or acknowledge its absence as a limitation.
8. Describe the synthesis process in greater detail, including how the integrated framework was derived.

**3. Is the statistical analysis and its interpretation appropriate?**

 This is a systematic literature review that employs narrative synthesis and thematic coding. No primary statistical analysis was conducted. The descriptive statistics presented in Table 2 (frequencies and percentages) are appropriate for summarizing study characteristics, but they are not inferential statistics and should not be interpreted as such. The authors correctly avoid causal or inferential claims based on these descriptive data.

**Recommendation:**

1. Clarify in the methods section that the review employs narrative synthesis and descriptive statistics only, and that no meta-analysis was conducted (and why).

**4. Are the conclusions drawn adequately supported by the results presented in the review?**

**Strengths:**

1. The conclusions are broadly consistent with the findings presented in Tables 3–5 and Figure 2.
2. The authors appropriately call for future research to move beyond isolated AI functions toward integrated, long-term studies of Human-AI Collaboration.
3. The proposed framework is clearly presented and grounded in the reviewed literature.

**Weaknesses and Required Revisions:**

1. **Causal language is used without sufficient evidence.** The authors state that "these processes are brought about by a variety of learning mechanisms" (p. 13). This is causal language that implies a deterministic relationship between mechanisms and outcomes. Given that the review synthesizes primarily correlational, quasi-experimental, and design-based studies—and does not conduct a meta-analysis or assess causal inference—this language is too strong. The authors should use more cautious phrasing such as "may be supported by," "appear to be facilitated by," or "are associated with."
2. **The claim that the framework "explains" how Human-AI Collaboration supports learning is overstated.** The framework is a conceptual synthesis, not an empirically validated causal model. The authors should frame it as a "heuristic" or "proposed" framework that requires empirical testing, rather than as an explanatory model.
3. **The statement "It seems to be more appropriate to treat the processes of students' conceptual understanding and their conceptual change as a matter of fact" (p. 13) is unclear and problematic.** The phrase "as a matter of fact" is ambiguous and appears to make a stronger claim than the evidence warrants. The authors should revise this passage to clearly state their intended argument—likely that conceptual understanding and conceptual change should be conceptualized as emergent, iterative processes rather than discrete instructional outcomes—and support this with evidence from the review.
4. **The conclusion does not adequately acknowledge the limitations of the review.** The authors should discuss:
  1. The narrow database coverage (Scopus only).
  2. The exclusion of pre-2023 studies.
  3. The lack of quality appraisal.
  4. The absence of preregistration.
  5. The potential for publication bias.
  6. The limited generalizability of the framework given the small number of studies (n = 28) and their concentration in certain science domains (e.g., physics = 32.14%).
5. **The framework's theoretical foundations are asserted rather than justified.** The authors state that the framework is based on constructivism, sociocultural theory, inquiry-based learning, and metacognitive theory (p. 12). However, they do not explain why these particular theories were chosen, how they were identified from the reviewed studies, or how they integrate with one another. A stronger theoretical justification is needed.
6. **The framework's components are not clearly distinguished.** The framework (Figure 2) presents five learning mechanisms (Adaptive Support, Knowledge Exploration and Construction, Reflection and Dialogue, Cognitive Conflict, Information Evaluation and Validation) and links them to learning outcomes. However, the relationships between these mechanisms—whether they are sequential, iterative, or interdependent—are not clearly explained. The authors should clarify the nature of these relationships and provide a more detailed narrative description of how the framework operates.
7. **The role of AI in the framework is underspecified.** The framework positions AI as a "learning partner," but does not clearly distinguish between different types of AI (e.g., LLMs, intelligent tutoring systems, machine learning) or explain how the framework applies across different AI technologies. Given the diversity of AI tools in the reviewed studies, the framework should account for this heterogeneity.

**Recommendations:**

1. Replace causal language with more cautious, associational phrasing.
2. Frame the framework as a proposed heuristic requiring empirical validation, not as an explanatory model.
3. Revise the "matter of fact" passage for clarity and accuracy.
4. Add a limitations section that acknowledges the review's methodological constraints.
5. Provide stronger justification for the theoretical foundations and clarify how the theories integrate.
6. Clarify the relationships between framework components and the role of different AI technologies.
7. Ensure that conclusions are explicitly tied to the evidence presented in Tables 3–5 and Figure 2.

**5. If this is a Living Systematic Review, is the 'living' method appropriate and is the search schedule clearly defined and justified?**

 The manuscript does not describe itself as a "Living Systematic Review," nor does it use this term in the title or text. Therefore, this question is not applicable. However, the authors may wish to consider whether a living systematic review approach would be appropriate given the rapidly evolving nature of AI in education. If they choose to adopt this approach, they should include "Living Systematic Review" in the title and clearly define the search schedule and update criteria.

**Summary of Required Revisions**

**Must Be Addressed (Scientific Soundness):**

1. **Provide an explicit operational definition of Human-AI Collaboration** and distinguish it from related constructs.
2. **Report inter-rater reliability** for screening and coding, or acknowledge its absence as a limitation.
3. **Include the PRISMA flow diagram** in the manuscript and report the number of records at each stage, with reasons for exclusion.
4. **Expand the database search** or justify the use of Scopus alone and acknowledge the limitation.
5. **Report whether the review protocol was preregistered** and provide registration details if available.
6. **Clarify the search date(s)** and whether the search was updated.
7. **Replace causal language with cautious, associational phrasing** in the conclusions.
8. **Revise the "matter of fact" passage** for clarity and accuracy.
9. **Add a limitations section** that acknowledges the review's methodological constraints.
10. **Provide a more detailed description of the thematic coding and synthesis process**, including who performed these tasks and how reliability was ensured.

**Should Be Addressed (Strengthening the Manuscript):**

1. **Justify the 2023–2026 publication window.**
2. **Support the "gaining momentum" claim with bibliometric or trend evidence.**
3. **Acknowledge related frameworks** and explain how the proposed framework extends or differs from them.
4. **Report quality appraisal of included studies** or acknowledge its absence as a limitation.
5. **Clarify the theoretical foundations** of the framework and how the theories integrate.
6. **Clarify the relationships between framework components** and the role of different AI technologies.
7. **Improve the grammatical and stylistic quality** of the manuscript (e.g., "a total of 28 empirical studies research were analyzed" should be corrected to "a total of 28 empirical studies were included in the analysis").
8. **Consider whether a living systematic review approach** would be appropriate given the rapidly evolving field.

**Conclusion**

 The manuscript addresses a timely and important topic, and the proposed integrated conceptual framework represents a valuable contribution to the literature. However, the review has several methodological limitations that must be addressed to ensure scientific soundness. The most critical issues are the lack of inter-rater reliability reporting, the absence of the PRISMA flow diagram in the manuscript, the narrow database coverage, and the use of causal language that exceeds the evidence. Addressing these issues will significantly strengthen the manuscript and enhance its contribution to the field.

Are the rationale for, and objectives of, the Systematic Review clearly stated?

Partly

Is the statistical analysis and its interpretation appropriate?

Not applicable

If this is a Living Systematic Review, is the ‘living’ method appropriate and is the search schedule clearly defined and justified? (‘Living Systematic Review’ or a variation of this term should be included in the title.)

Not applicable

Are sufficient details of the methods and analysis provided to allow replication by others?

No

Are the conclusions drawn adequately supported by the results presented in the review?

Partly

Reviewer Expertise:

Educational Technology; Artificial Intelligence in Education; Science Education; Systematic Review Methodology; Learning Sciences

I confirm that I have read this submission and believe that I have an appropriate level of expertise to confirm that it is of an acceptable scientific standard, however I have significant reservations, as outlined above.

# Reviewer report: Reviewer response for version 1

**DOI:** 10.5256/f1000research.206380.r515589

**Published:** 2026-09-02

**Authors:** Nikolaos Pellas

**Affiliations:** 1 Aristotle University of Thessaloniki, Thessaloniki, Greece

Overall assessment

 The text addresses a timely and potentially valuable topic, and the attempt to move beyond tool-specific evaluations toward **learning mechanisms underlying Human–AI collaboration** is a clear strength. The proposed integrated conceptual framework could make a useful contribution. However, several aspects require clarification, particularly the definition of Human–AI collaboration, the distinction between conceptual understanding and conceptual change, the derivation and validation of the proposed mechanisms, and the methodological reporting of the systematic review.

 Major comments and specific questions

**1. Clarify the central concept of “Human–AI Collaboration.”**

 The manuscript repeatedly uses “Human AI Collaboration,” but the concept does not appear to be sufficiently operationalized.

**Question:**

 How do the authors define *Human–AI collaboration* in this review, and what criteria were used to distinguish genuine collaboration from more conventional uses of AI, such as AI-assisted tutoring, automated feedback, question answering, or content generation?

**Suggestion:**

 Provide an explicit conceptual definition and inclusion criteria. It would also be useful to explain what makes the interaction *collaborative* rather than simply *AI-supported learning*.

**2. The opening claim needs stronger support.**

 The statement that Human–AI collaboration in science education “is gaining momentum” is plausible, but it should be supported by recent evidence.

**Question:**

 What evidence demonstrates that Human–AI collaboration is specifically gaining momentum, rather than AI in education more generally?

**3. There is a conceptual ambiguity between conceptual understanding and conceptual change.**

 The manuscript treats these concepts as closely related, but they are not necessarily interchangeable. Conceptual understanding may involve constructing or deepening scientifically accurate representations, whereas conceptual change often concerns restructuring or replacing prior conceptions.

**Question:**

 How are *conceptual understanding* and *conceptual change* operationally defined and distinguished in the review? Did the included studies explicitly measure conceptual change, or did the authors infer conceptual change from improvements in conceptual understanding?

 This distinction is particularly important because the manuscript later concludes that these processes are “brought about by a variety of learning mechanisms.”

**4. The reported number of learning mechanisms is internally inconsistent.**

 The abstract/text states that “five mechanisms supporting the students’ conceptual change were identified,” followed by “Five interconnected learning mechanisms were derived.” However, the manuscript also states that **six roles of AI** were identified.

**Question:**

 Are there exactly five learning mechanisms and six AI roles? What is the relationship between these two categories?

**Suggestion:**

 A concise table showing **AI role → learning mechanism → expected effect on conceptual understanding/conceptual change → supporting studies** would substantially improve clarity.

**5. The derivation of the framework requires more methodological transparency.**

 The manuscript states that an integrated conceptual framework was derived from the included studies and is based on constructivism, sociocultural theory, inquiry-based science learning, and metacognitive theory.

**Questions:**

 How were the five mechanisms inductively or deductively identified?

 Were the theoretical perspectives specified before data extraction, or were they introduced after the patterns emerged from the literature?

 How did the authors avoid imposing these theories onto the findings?

 This is important because the framework appears to combine several theoretical traditions that have different assumptions about learning.

**6. The theoretical integration needs further justification.**

 The framework draws simultaneously on constructivism, sociocultural theory, inquiry-based learning, and metacognitive theory. This could be a strength, but currently the rationale for combining them is underdeveloped.

**Question:**

 What unique explanatory contribution does each theoretical perspective make to the proposed framework, and why are these four perspectives necessary rather than alternative theoretical lenses?

**7. The phrase “AI functions as a learning partner” may be too strong.**

 The wording suggests agency and reciprocity on the part of AI. Many AI systems used in education may provide responses or feedback without engaging in genuinely reciprocal collaborative processes.

**Question:**

 What evidence from the included studies supports characterizing AI as a *learning partner* rather than as a pedagogical tool, tutor, scaffold, or conversational agent?

**Suggestion:**

 The authors could distinguish between **functional partnership** and **human-like agency** to avoid anthropomorphic interpretations.

**8. Clarify the scope of the evidence base.**

 The review is based exclusively on Scopus.

**Question:**

 Why was Scopus selected as the sole database? Were Web of Science, ERIC, Education Research Complete, PsycINFO, IEEE Xplore, or other relevant databases considered?

 A single-database search may introduce database-selection bias, particularly in an interdisciplinary area spanning education, learning sciences, computer science, and AI.

**9. The PRISMA statement needs methodological detail.**

 The statement “In line with the PRISMA 2020 guidelines” is insufficient by itself.

**Questions:**

 Was the review protocol preregistered?

 Was a PRISMA 2020 flow diagram provided?

 How many records were identified, screened, excluded, and included?

 What were the principal exclusion reasons?

 For a systematic review, these details are important for reproducibility.

**10. The wording concerning the 28 studies should be corrected.**

 The sentence “a total of 28 empirical studies research were analyzed” is grammatically incorrect and conceptually awkward.

 A clearer formulation would be:

 “A total of 28 empirical studies were included in the analysis.”

 The phrase “These studies of empirical research” is similarly repetitive.

**11. The date of the search needs clarification.**

 The manuscript states that the Scopus search was “last updated on 15 June 2026.”

**Question:**

 Does this mean the original search was conducted on that date, or was the search initially conducted earlier and subsequently updated? If it was an update, when was the original search performed?

 This distinction is important for reporting the search strategy accurately.

**12. The publication period raises an important question about the evidence base.**

 The included studies were published between 2023 and 2026.

**Question:**

 What search and eligibility criteria resulted in no eligible empirical studies before 2023? Given the long history of intelligent tutoring systems, adaptive learning, computer-supported collaborative learning, and conversational agents, the authors should explain whether earlier research was explicitly excluded or whether it did not satisfy the Human–AI collaboration criteria.

**13. “Conceptual change” appears to be treated partly as an outcome and partly as a process.**

 The manuscript refers to conceptual understanding and conceptual change as processes, but elsewhere discusses mechanisms “supporting” them.

**Question:**

 Does the framework conceptualize conceptual change as an outcome, a cognitive process, or both? How is this represented in the framework?

 A clear distinction between **AI affordances, interaction processes, learning mechanisms, cognitive processes, and learning outcomes** would strengthen the model considerably.

**14. The evidence supporting causal language should be examined carefully.**

 The statement:

 “these processes are brought about by a variety of learning mechanisms”

 appears causal.

**Question:**

 Do the included studies provide sufficient experimental or longitudinal evidence to conclude that these mechanisms *bring about* conceptual change, or do they primarily indicate associations and plausible explanatory mechanisms?

 Given that this is a literature review, more cautious language such as *“may be supported by”*, *“appear to be facilitated by”*, or *“are associated with”* may be appropriate unless causal evidence is explicitly available.

**15. The final interpretation appears stronger than the evidence presented.**

 The statement:

 “it seems to be more appropriate to treat the processes of students’ conceptual understanding and their conceptual change as a matter of fact”

 is unclear and potentially problematic.

**Question:**

 What does “as a matter of fact” mean in this context? Is the intended argument that conceptual understanding and conceptual change should be conceptualized as **emergent learning processes rather than predefined instructional outcomes**?

 The authors should revise this passage because the current wording is difficult to interpret and appears stronger than the evidence warrants.

Are the rationale for, and objectives of, the Systematic Review clearly stated?

Partly

Is the statistical analysis and its interpretation appropriate?

Not applicable

If this is a Living Systematic Review, is the ‘living’ method appropriate and is the search schedule clearly defined and justified? (‘Living Systematic Review’ or a variation of this term should be included in the title.)

Partly

Are sufficient details of the methods and analysis provided to allow replication by others?

No

Are the conclusions drawn adequately supported by the results presented in the review?

Partly

Reviewer Expertise:

Educational Technology

I confirm that I have read this submission and believe that I have an appropriate level of expertise to confirm that it is of an acceptable scientific standard.
